# Inflow from a Cardiopulmonary Assist System to the Pulmonary Artery and Its Implications for Local Hemodynamics—a Computational Fluid Dynamics Study

**DOI:** 10.1007/s12265-022-10349-3

**Published:** 2023-01-20

**Authors:** Kristin Hugenroth, Felix Krooß, Flutura Hima, Lasse Strudthoff, Rüdger Kopp, Jutta Arens, Sebastian Kalverkamp, Ulrich Steinseifer, Michael Neidlin, Jan Spillner

**Affiliations:** 1 Department of Cardiovascular Engineering, Institute of Applied Medical Engineering, Medical Faculty, RWTH Aachen University, Aachen, Germany; 2 Department of Thoracic and Cardiovascular Surgery, Medical Faculty, University Hospital, RWTH Aachen University, Aachen, Germany; 3 Department of Intensive Care Medicine and Intermediate Care, Medical Faculty, University Hospital, RWTH Aachen University, Aachen, Germany

**Keywords:** CFD simulation, In silico, Graft connection, ECMO, Artificial lung, Retrograde flow, Lung perfusion

## Abstract

When returning blood to the pulmonary artery (PA), the inflow jet interferes with local hemodynamics. We investigated the consequences for several connection scenarios using transient computational fluid dynamics simulations. The PA was derived from CT data. Three aspects were varied: graft flow rate, anastomosis location, and inflow jet path length from anastomosis site to impingement on the PA wall. Lateral anastomosis locations caused abnormal flow distribution between the left and right PA. The central location provided near-physiological distribution but induced higher wall shear stress (WSS). All effects were most pronounced at high graft flows. A central location is beneficial regarding flow distribution, but the resulting high WSS might promote detachment of local thromboembolisms or influence the autonomic nervous innervation. Lateral locations, depending on jet path length, result in lower WSS at the cost of an unfavorable flow distribution that could promote pulmonary vasculature changes. Case-specific decisions and further research are necessary.

**Graphical Abstract:**

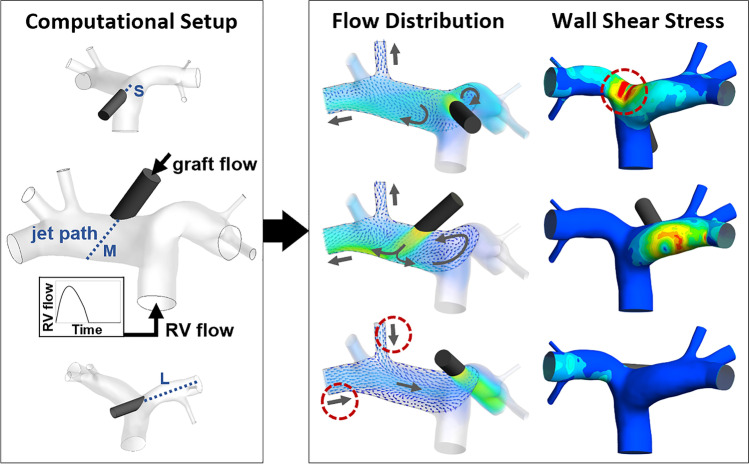

**Supplementary Information:**

The online version contains supplementary material available at 10.1007/s12265-022-10349-3.

## Introduction

Lung support by extracorporeal membrane oxygenation (ECMO) is an established treatment option for patients with acute respiratory failure or as a bridge to lung transplant. For respiratory support, the ECMO system is connected to the patient with two single-lumen cannulas or one dual-lumen cannula in a veno-venous configuration (drainage from and reinfusion to the venous system, VV-ECMO). Access points in adult patients are the femoral and jugular veins, and the cannulas are advanced to the central vessels to enable high flow rates. The use of cannulas is, however, accompanied by drawbacks, which make long-term application of VV-ECMO challenging, for example, complications at the connection site of the extracorporeal circuit [1–3] and because of poor mobilizability of patients [4, 5]. Since the treatment is limited to short term, it is of limited use for managing chronically ill patients. However, with an epidemiological increase of chronic respiratory failure and stagnant availability of donor organs, the long-term use of ECMO is inevitable. Therefore, new connection options need to be developed to avoid the risks associated with cannulation and to provide mobility and a better quality of life for the patients.

Criteria for the connection are sufficient blood flow for extracorporeal oxygenation, long-term stability of the connection site, and low risk for complications, like hemolysis, thrombus generation, and occlusion of cannula or vessels.

Long-term stability and low complication risk could be achieved by using graft anastomoses instead of cannulas for the vessel access. Graft anastomoses are a widely used connection method in various indications. An end-to-side anastomosis to the PA is already an established procedure in the use of right ventricular assist devices (RVADs) [6, 7] and is equally conceivable for patients with long-term pulmonary support.

Sufficient blood flow can only be achieved by connection to the central vessels, as they provide the highest flow rates throughout the body [2]. Possible central connection sites are the heart, intrathoracic aorta, and pulmonary artery [8]. Access to these vessels is commonly achieved by (median) sternotomy. This highly invasive surgical procedure poses a high risk for critically ill ECMO patients. A minimally invasive approach can improve the healing process [9] and therefore make ECMO connection to the central vessels a viable option. From the central vessels, the right atrium can be used for drainage and the pulmonary artery (PA) for reinfusion to facilitate a minimally invasive connection procedure. A lateral connection to the PA may be necessary to allow for a minimally invasive approach to access its extrapericardial parts from between the ribs.

The perfusion of non-native blood flow into a blood vessel represents a major interference with the local flow conditions in the respective vessel. Substantial changes to the flow conditions, in turn, can lead to permanent damage, especially when present for a longer period of time [7]. Computational fluid dynamics (CFD) has proved to be a valuable tool to investigate the flow in blood vessels, providing data of high spatial and temporal resolution, for example, of the aorta [10–12] or the PA [13–15]. The changes induced by the inflow jet from a graft [16–18] or cannula [11, 19, 20] have been investigated, showing, among others, nonphysiologic flow patterns and high wall shear stress (WSS) in the inflow region. The reinfusion of graft flow to the PA has, however, not yet been investigated. In this study, we examine the local flow conditions resulting from this reinfusion configuration with the aim of analyzing the influence of anastomosis location and graft flow on flow distribution to the PA branches and WSS. We include lateral anastomosis locations, to explore the potential for a minimally invasive approach. With this study, we intend to reveal potential risks as well as specific aspects that have to be considered when creating a graft anastomosis to the PA.

## Methods

We performed CFD simulations to compare different grafting scenarios with varying anastomosis locations, graft orientations, and levels of circulatory support. The results were analyzed regarding flow distribution to the PA branches and regarding wall shear stress (WSS).

**Fig. 1:**
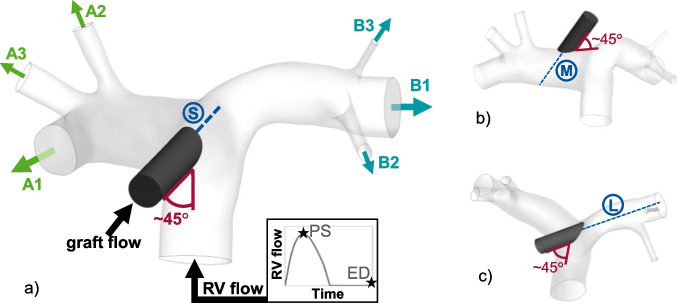
Simulation models. (**a**) Central graft anastomosis, short jet path (S). (**b**) Lateral graft anastomosis, medium jet path (M). (**c**) Lateral graft anastomosis, long jet path (L). Arrows indicate physiological flow direction at the boundaries. The embedded graph shows the course of the flow rate at the right ventricle (RV) inlet over one cardiac cycle and indicates the points of peak systole (PS) and end-diastole (ED), which are later used for evaluation purposes. A1, B1: main branches on side A and side B. A2, A3, B2, B3: side branches on side A and side B

The pulmonary artery (PA) anatomy used in this study was extracted from CT imaging data of a healthy male human volunteer using Mimics (Materialise NV, Leuven, Belgium). From this, four 3D models were generated using 3-matic (Materialise NV, Leuven, Belgium): one of a physiological anatomy without graft and three with a graft anastomosis in different locations and orientations (see Table 1 and Fig. 1).

**Table 1:** Boundary conditions of the simulations

Simulation model	Physiological	S		M		L	
Graft	no	yes		yes		yes	
Anastomosis location	-	central		lateral		lateral	
Graft extension length	-	short		medium		long	
Graft flow (L/min)	-	2.5	5.0	2.5	5.0	2.5	5.0
Right ventricle flow (L/min)	5	2.9	0.8	2.9	0.8	2.9	0.8

Anastomosis location and graft orientation produce different lengths of the extension of the graft to the vessel wall, indicated by the blue dashed lines in Fig. 1. The length of the graft extension corresponds with the maximum available length of an inflow jet from the graft. Therefore, the three scenarios with graft anastomosis will be referred to as the “short jet path” (S), “medium jet path” (M), and “long jet path” (L) in the following. Since the main difference between the two lateral positions is not the respective side of the PA but the jet path, and to avoid potential invalid conclusions, we will refer to the sides of the PA by “side A” and “side B” in the following, where “A” refers to the right branches of the PA and “B” to the left branches (see Fig. 1).

The graft was idealized as a straight tube with a diameter of 10 mm and length of approximately 30 mm. Its alignment to the vessel was chosen based on two criteria:

1. Approximately 45° angle to the vessel and pointed in the direction of the native flow (angle indicated in red in Fig. 1). Recently, this has been shown to be beneficial regarding local hemodynamics [7]
2. Feasibility of minimally invasive access

The simulation meshes were generated using ICEM (Ansys Inc., Canonsburg, PA, USA) from tetrahedral elements (maximum size 1 mm) and 8 prism layers (initial height 0.04 mm, height ratio 1.2); see Online Resource 1, S1. A mesh sensitivity study was conducted that determined sufficient independence for a mesh of 945,000 elements; see Online Resource 1, S2.

Simulations were conducted with Fluent (Ansys Inc., Canonsburg, PA, USA). To account for turbulent effects, we chose the Transitional SST turbulence model, since Reynolds numbers in the transitional range were estimated (see Online Resource 1, S3). The blood was simulated simplified as a Newtonian fluid with a density of 1056.4 kg/m^3^ and dynamic viscosity of 3.6 mPa s. Pulsatile inflow boundary conditions from the right ventricle were derived from a lumped parameter simulation model, which was adjusted to include the drainage and infusion of an ECMO circuit at the right atrium and pulmonary artery, respectively. ECMO flows of 2.5 L/min and 5.0 L/min were simulated with the lumped parameter model and applied as the inflow boundary condition at the graft inlet in the CFD model. Following Reynolds number estimations, the turbulence intensity at the graft inlet was set to 0% in the 2.5 L/min cases and to 5% in the 5.0 L/min cases. Similarly, it was set to 5% for the right ventricular inflow in all cases. Pressure of 20 mmHg and 0% turbulence intensity were assigned to all other boundaries. For details on the boundary conditions, refer to Online Resource 1, S3. Convergence was ensured by allowing a maximum residuum of 0.0001 and cycle dependency was controlled by monitoring mass flows and pressures at all in- and outlets.

Post processing was performed using CFD-Post (Ansys Inc., Canonsburg, PA, USA). The presented results refer to the eighth simulated heart cycle, for which variations of mass flow and pressures at the boundaries compared to the previous cycle were below 2%. Some evaluations refer to the results at peak systole or end-diastole. These points were chosen, because at peak systole the impact on the wall can be expected to be highest due to the maximum flow rate (a maximum ventricular flow is superimposed on the constant graft flow) and at end-diastole the effects caused by the jet flow can be best observed (least influence by the ventricular flow).

## Results

With the CFD simulations, we investigated two major aspects: flow distribution to the PA branches and WSS on the vessel wall.

### Flow Distribution

**Fig. 2:**
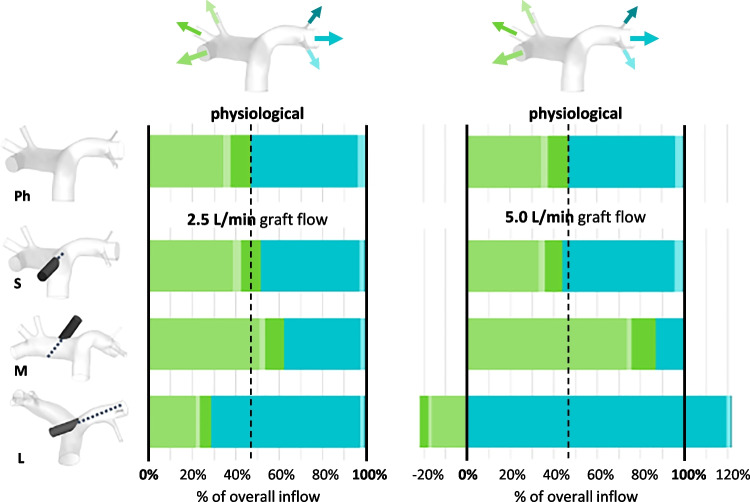
Flow distribution to the PA branches in percentage of the overall inflow (graft + ventricular flow) at 2.5 L/min (left) and 5.0 L/min (right) graft flow. Negative values indicate retrograde flow at the respective branches. Ph, physiological setup without graft; S, short jet path; M, medium jet path; L, long jet path. Dashed line: physiological distribution to left and right sides

The flow rates at the pulmonary artery branches differ depending on graft flow rate and anastomosis location. We compared the difference by integrating the flow rate at each branch over one cardiac cycle to gain the overall flow through the respective branch during one cycle. In Fig. 2, the overall flows through the branches are displayed as percentage of overall inflow (graft flow plus ventricular flow).

For the physiological simulation setup without a graft, the distribution between side A and side B is fairly even (A: 46.83% of overall flow, shades of green in Fig. 2; B: 53.17% of overall flow, shades of teal). At 2.5 L/min graft flow, changes in the distribution are apparent mainly for the setups with the lateral anastomosis locations. The proportion of the flow towards the side, on which the graft is located, is increased (graft on side A, medium jet path: 62.33% of overall flow to side A, 37.67% to side B; graft on side B, long jet path: 28.82% of overall flow to side A, 71.18% to side B).

The deviation from the physiological distribution is more pronounced at 5.0 L/min graft flow and again for the setups with the lateral anastomosis locations. With the graft on side A and medium jet path, the flows to side B are very low. With the graft on side B and long jet path, the flows to side A are even reversed (see negative values in Fig. 2). This indicates that over the cardiac cycle, the total amount of blood flowing in retrograde direction is greater than the amount flowing in an anterograde direction in the PA branches on side A. At the same time, flow to side B is approximately 2.5-fold higher than detected in the simulation with physiological conditions.

**Fig. 3:**
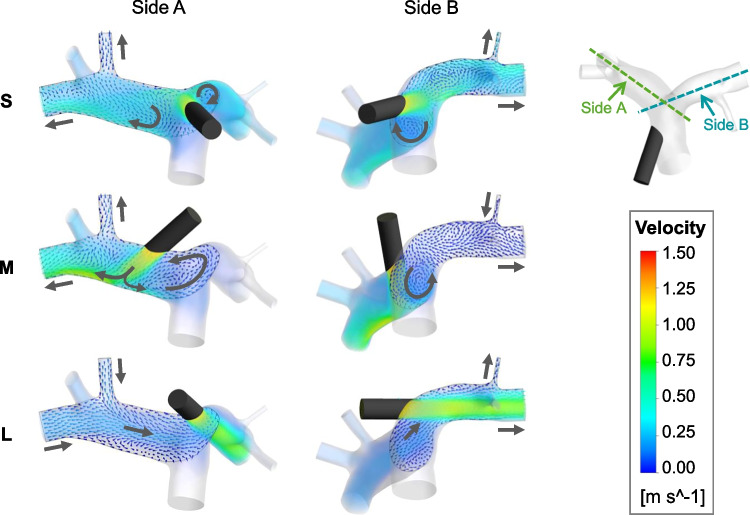
Flow field in the pulmonary artery at end-diastole with 5.0 L/min graft flow visualized by vectors on two planes (view from side A, view from side B; plane locations depicted in the top right image) and transparent volume rendering. Gray arrows indicate flow direction at the branches and major flow patterns. S, short jet path; M, medium jet path; L, long jet path. Note the backflow in the images “M, Side B” and “L, Side A”

We investigated the underlying mechanisms by visualizing the flow field. Fig. 3 shows the flow patterns at end-diastole with 5.0 L/min graft flow, which is the scenario in which the impact of the graft flow on the flow pattern is most pronounced. The inflow jet from the graft generates complex flow patterns in the pulmonary artery, including larger vortices near the bifurcation. Low-velocity backflow is observed with the graft on side A and medium jet path through a contralateral PA branch. Similarly, backflow occurs with the graft on side B and long jet path through the main and a side branch on the opposite side, but at a slightly higher velocity. Note that the view planes and the point in the cardiac cycle were only chosen for visualization purposes; backflow was also observed through other branches at other time steps, which can be observed in the Online Resource 2.

### Wall Shear Stress

**Fig. 4:**
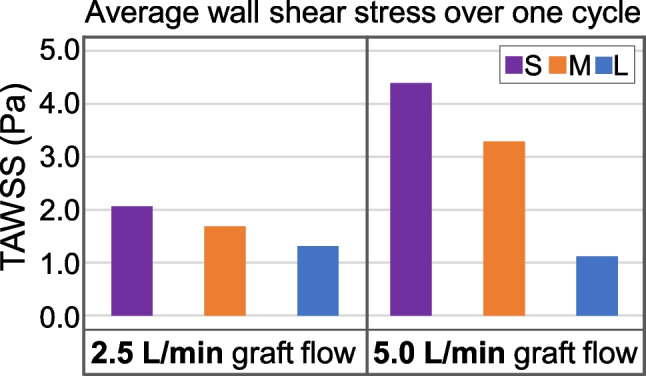
Time-averaged wall shear stress (TAWSS) on the pulmonary artery wall for 2.5 L/min and 5.0 L/min graft flow. S, short jet path; M, medium jet path; L, long jet path

The WSS on the vessel wall differs depending on graft flow rate and length of the jet path. The average WSS over the cardiac cycle (time-averaged WSS: TAWSS) was analyzed to estimate the impact on the vessel wall. As shown in Fig. 4, the TAWSS is markedly higher and the difference between the three jet path cases is more pronounced at 5.0 L/min compared to that at 2.5 L/min graft flow (see also Online Resource 1, S4–S6). At both flow rates, the setup with the central anastomosis location and short jet path results in the highest TAWSS values (2.1 Pa and 4.4 Pa), while the setup with the lateral anastomosis location and long jet path results in the smallest values (1.3 Pa and 1.1 Pa). The same tendency can be observed when analyzing the maximum WSS values (see Online Resource 1, S4).

**Fig. 5:**
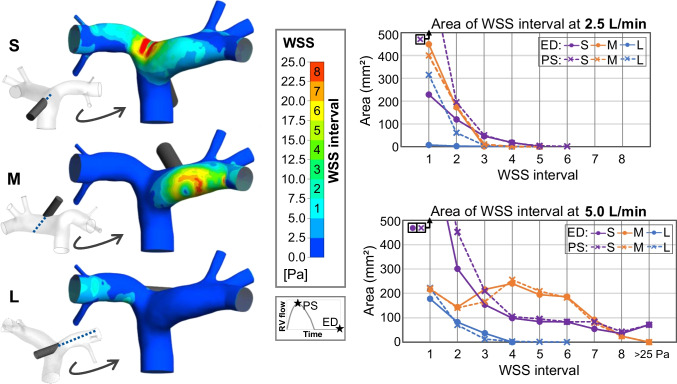
Wall shear stress (WSS) on the pulmonary artery wall. Left: WSS contours at end-diastole (ED) and 5.0 L/min graft flow, posterior view. Small images to the left show anterior view for better differentiation. Center: legend of WSS values and corresponding WSS intervals; below it, a small graph of the right ventricle (RV) flow to illustrate the points of peak systole (PS) and end-diastole (ED) used for the analysis. Right: areas of the respective WSS intervals at end-diastole (ED) and peak systole (PS) for 2.5 L/min (top) and 5.0 L/min (bottom) graft flow. The *y*-axis was clipped at 500 mm^2^ for enhanced clarity, not displayed are “S, PS” at 2.5 L/min: 791.5 mm^2^, “S, ED” at 5.0 L/min: 835.4 mm^2^, and “S, PS” at 5.0 L/min: 995.8 L/min. S, short jet path; M, medium jet path; L, long jet path

Figure 5, left, shows the regions in which the WSS is elevated. With central anastomosis location and short jet path, especially the PA bifurcation opposite of the graft anastomosis is affected. The progression over time can be observed in the Online Resource 3. With the lateral location and medium jet path, it is the PA wall at the point where the inflow jet hits the wall. With the lateral location and long jet path, WSS values along the PA branch, in which the jet is directed, are elevated.

We evaluated the distribution between high and low WSS values for a more comprehensive analysis of the resulting WSS. The PA wall was partitioned into arbitrary WSS intervals, each of which covered a range of 2.5 Pa (see the example in Fig. 5, left, and corresponding legend). The area of these intervals was then evaluated to assess the WSS distribution (see Fig. 5, right).

At 2.5 L/min graft flow, the distribution of WSS below 10 Pa (WSS intervals 1 and 2) varies between end-diastole and peak systole for the small and long jet path (see Fig. 5, top right). The area in which the WSS exceeds 10 Pa (WSS interval 3 and above) is notably larger with the short jet path compared to the medium and long jet path. However, with none of the three configurations, the WSS exceeds 20 Pa.

In comparison, the WSS areas are markedly larger at 5.0 L/min graft flow and differences between end-diastole and peak systole are less pronounced (see Fig. 5, bottom right). Two main observations can be stated regarding the WSS distribution at 5 L/min graft flow: Firstly, the areas of high WSS are clearly smallest with the long jet path. Secondly, the distribution of WSS values is very different between the configurations with short and medium jet path. Elevated WSS values (below 7.5 Pa) are more prevalent with the short jet path compared to the medium one, high WSS values (12.5–20 Pa) are more prevalent with the medium jet path, and very high WSS values (over 22.5 Pa) are again more prevalent with the short jet path.

## Discussion

In this study, we investigated the effect of returning blood to the PA through a graft anastomosed to the PA wall. We analyzed the influence of anastomosis location and graft orientation, which define the length of the inflow jet, and graft flow rate on the flow distribution to the PA branches and on the WSS on the vessel wall. Jet path length and flow rate both showed a major impact on flow distribution and WSS, but the indications for which anastomosis location and jet path length could be beneficial are contradictory.

### Flow Distribution

The flow distribution was investigated because we suspected a strong effect of the jet flow on the local flow field. Jet flow has shown to cause retrograde flow in adjacent branches in previous studies [21]. In this context, hypoperfusion or retrograde flow in the pulmonary arteries could potentially lead to pulmonary vasculature changes like endothelial activation or clot generation. Therefore, a physiological distribution, which entails a fairly even distribution between the left and right pulmonary arteries, would be desirable.

Our results show that the flow distribution varies depending on the anastomosis location and jet path length at 2.5 L/min to some extent: with the central anastomosis location and short jet path, the distribution is close to the physiological state, while the lateral anastomosis locations result in increased flow to the branches on the side of the graft anastomosis (cf. Fig. 2). With the central anastomosis to the pulmonary trunk, the graft flow hits the opposite wall at the bifurcation and splits, supplying both sides of the pulmonary artery fairly equally. With the lateral anastomosis locations and the graft orientation being in the direction of the native flow however, the graft flow adds to the flow from the right ventricle on one side more than on the other.

A much higher influence of the inflow jet is observed at 5.0 L/min graft flow. In these scenarios, the flow from the right ventricle is much lower than that at 2.5 L/min (0.8 L/min vs. 2.9 L/min). This then results in the graft flow having a much higher impact on the overall flow field in the pulmonary artery. With the central anastomosis location, the distribution to the adjacent arteries is still close to physiological for the reason stated above. With the lateral anastomosis location and medium jet path length, the flow to the branches on the same side as the graft is greatly increased, while the flow to the contralateral branches is equally reduced.

Visualization of the flow field reveals the underlying mechanisms: the jet flow from the graft results in a suction effect that slows down and partially reverses the flow in the contralateral branch of the PA (cf. Fig. 3). This effect is mitigated in the scenario of the medium jet path: the jet hits the vessel wall, which causes the flow to be partially redirected against the native flow direction, generating a vortex at the PA bifurcation. With the scenario of the long jet path, however, the vortex at the PA bifurcation is not observed, since the jet does not hit the wall anywhere close to the bifurcation. Therefore, the suction effect is stronger in the contralateral branch, completely reversing the flow.

Overall, the central anastomosis location appears to be most beneficial regarding the flow distribution to the PA branches, and the lateral location with the long jet path leads to undesirable effects.

### Wall Shear Stress

Wall shear stress was investigated because high values have shown to be associated with inflammation and debris detachment [22]. In the PA, detachment of local thromboembolisms and an increase in sympathetic tone are additional risks, the latter being promoted by impact on the sensory fibers for sympathetic innervation of the pulmonary vessels [23–26]. Therefore, lower WSS values and small areas of elevated WSS are desirable, especially in sensitive regions of the PA. WSS analysis is used as a qualitative comparison between the presented cases.

Similar to the flow distribution, the WSS is influenced by the jet flow hitting the wall. With the central anastomosis location and short jet path, the jet flow velocity is barely decelerated when it hits the vessel wall (cf. Fig. 3, S). High near-wall velocity leads to high WSS, which is observed in the impingement region (cf. Fig. 5, top). With the medium jet path, the distance between the anastomosis location and the point where the jet hits the wall is larger; therefore, the jet flow is more decelerated and causes lower maximum WSS. However, the jet hits the wall at a flatter impingement angle, generating a larger region of elevated near-wall velocity (cf. Fig. 3, side A view, M) and resulting in a larger area of high WSS (cf. Fig. 5). The scenario of the long jet path results in overall low WSS and small areas of elevated WSS. The reason is that the jet path is parallel to the vessel wall, only slightly increasing the near-wall velocity (cf. Fig. 3, side B view, L).

When comparing the scenarios of 2.5 L/min with those of 5.0 L/min graft flow, the latter exhibit mostly higher WSS values and larger areas of high WSS (cf. Fig. 4 and Online Resource 1, S4–S6). The only exception is the average WSS values in the scenario with the long jet path. Here, a higher value can be observed at peak systole with 2.5 L/min graft flow compared to 5.0 L/min graft flow. In this case, the low WSS induced by the jet flow plays a minor role compared to the WSS induced by the ventricular flow. Another observation when comparing 2.5 L/min and 5.0 L/min scenarios regarding WSS values correlates with the findings regarding flow distribution: the difference between the three scenarios with different jet path length is more pronounced at 5.0 L/min compared to 2.5 L/min. This can again be attributed to a change of the ratio between graft flow and ventricular flow (2.5 L/min and 2.9 L/min vs. 5.0 L/min and 0.8 L/min), which indicates that the influence of the jet flow on the local flow field is higher in the 5.0 L/min scenarios.

Overall, and in contrast to the results from the flow distribution analysis, the lateral anastomosis location with the long jet path appears to be most beneficial, while the central location with short jet path leads to the highest WSS values.

## Combined Discussion

The previous sections lead to contradictory results as to which anastomosis location and jet path length may be better suited for an inflow graft on the PA. In terms of flow distribution, the central anastomosis location seems most suitable and the lateral location with a long jet path seems least suitable, while WSS-wise it is the other way around. An intermediate option can be a good solution, with a jet path that is longer compared to the central location, but hits the wall not too far from the PA bifurcation. This could be accomplished with a lateral inflow graft location, enabling access using minimally invasive techniques.

The results clearly show that it is worth considering patient-specific details before placing a graft or cannula, which returns blood to the PA. The following questions should be addressed:

- What are the expected flow rates? At 2.5 L/min, the effects were considerably less pronounced and might therefore not be as relevant at low or medium support.
- What are the specific features of the patient’s anatomy, and how strong are the curvatures of the main PA branches? Depending on this, the location and orientation of a lateral graft placement may favor a jet path that does not hit the wall close to the PA bifurcation and that may therefore lead to stagnation and reversed flow in the contralateral PA branch.
- Has buildup or thromboembolism of the major PAs been observed in the patient? A jet hitting the respective region might cause detachment of debris and promote chronic thromboembolic disease [27].

Every patient is different, and we recommend a pre-operative CT scan to understand the individual situation and thereby gain a basis for decision-making.

## Limitations and Future Work

We conducted this study to gain first insights into the local hemodynamics and the influence of anastomosis location, jet path length, and graft flow. We therefore chose CFD simulations, which provide relatively quick results with high temporal and spatial resolution, while enabling defined changes to a consistent anatomy.

However, CFD cannot mimic biological processes and a possible reaction of the body to the induced changes. This is especially relevant for the observed retrograde flow from the PA branches. Autoregulation mechanisms could react to the hemodynamic changes, possibly attenuating or overcoming the effects of retrograde flow and/or blood stagnation [28]. Previous CFD studies of other vessels included more sophisticated lumped parameter models at the outflow boundaries [13, 21]. This method can be applied to the current problem to mimic autoregulation reactions and to generate more realistic boundary conditions at the PA branches.

Validation of the current simulation setup without autoregulation can be done by in vitro experiments, like particle image velocimetry or ink tests, using the same geometries as in the present study. In vitro experiments of this setup will be very challenging and complex if they account for autoregulation mechanisms. Both in silico and in vitro results will need to be validated by in vivo data because they rely on assumptions regarding autoregulation. Obtaining correct in vivo data is desirable, but it is complicated by the fact that anesthetics influence autoregulation mechanisms, which excludes acute animal trials, only leaving elaborate and costly chronic animal trials. They furthermore depend highly on a good anatomical and physiological transferability of the animal model. Ideally, imaging data as well as flow and pressure measurements from in vivo experiments are then used in CFD simulations and in vitro tests for validation and adaption of the boundary conditions.

Studying more PA anatomies using the same approach of e.g. three jet path lengths can help verify the aforementioned conclusions and can add to the predictive value of the presented results. It can be expected that with variations in the PA anatomy (change in bifurcation angulation, vessel diameter, etc., or anatomical abnormalities) the jet path lengths vary when using the same anastomosis locations. Consequently, anastomosis location and orientation may need to be adapted. Similarly, studying more anastomosis locations, graft orientations, and flow rates as well as additional conditions like increased heart rate, pulmonary hypertension, or pulmonary regurgitation on the same anatomy will generate a larger data basis, which enables a more differentiated assessment of the hemodynamic changes introduced by the jet flow.

In this study, blood was modeled as a Newtonian fluid. In fact, it has shear-thinning non-Newtonian properties, which can be attributed mostly to the erythrocytes in the blood and their mechanical properties. The shear dependency is especially relevant at low shear rates and can affect WSS estimation in these regions [29]. Models have been developed to account for the shear-thinning behavior of the blood and further investigations should include the application of these models.

As mentioned before, taking patient-specific details into account when planning a graft anastomosis to the PA can avoid iatrogenic complications. In this context, patient-specific simulations can aid in decision-making. It would be conceivable to simulate different anastomosis locations and graft orientations incorporating the patient’s anatomy prior to the procedure. A quantitative and qualitative evaluation, as presented in this publication, facilitates a direct comparison of different options. A further step would be a simulation-based, automated iteration of anastomosis locations and graft orientations, which suggests an optimal option to the clinician.

## Conclusion

Our results highlight that returning blood via a graft or cannula to the pulmonary artery (PA) has a major impact on local hemodynamics. These implications are of particular interest, when aiming for a minimally invasive connection method, which will probably use a lateral access. Retrograde flow in the adjacent segmental branches as well as high wall shear stress on the vessel wall may occur, especially at high flow rates. These effects are highly dependent on the inflow jet and its path which is determined by the anastomosis location and graft orientation in combination with the patient-individual anatomy. The jet flow from the graft should neither be directed straight to the wall (risk of high wall shear stress) nor be directed into one PA branch without hitting the wall (risk of retrograde flow in the contralateral PA branches). Overall, the study underlines that the anastomosis location and graft orientation should be chosen carefully to avoid complications caused by unfavorable hemodynamics.

## Supplementary Information

Below is the link to the electronic supplementary material.

Supplementary file1 Online Resource 1: OR_1_SupplementaryMaterial.pdf. The Supplementary Material contains information regarding meshing parameters, mesh convergence analysis and simulation setup as well as additional figures of the wall shear stress on the vessel wall. (PDF 465 KB)

Supplementary file2 Online Resource 2: OR_2_Video_L_FullSupport_Flow.wmv. The video shows the flow in the pulmonary artery for the setup with the long jet path at full support for one cycle. It illustrates the in- and outflow at the boundaries, highlighting the retrograde flow at some pulmonary artery branches. (WMV 2019 KB)

Supplementary file3 Online Resource 3: OR_3_Video_S_Flow_WSS.wmv. The video shows both flow and wall shear stress for the setup with the short jet path at both partial and full support. It illustrates the high wall shear stress at the region, where the graft inflow jet hits the vessel wall, which arises in the case of full support. (WMV 3265 KB)

## Data Availability

All underlying data of the current study is available from the corresponding author on reasonable request.

## Abbreviations

CFD: Computational fluid dynamics
ECMO: Extracorporeal membrane oxygenation
PA: Pulmonary artery
WSS: Wall shear stress

## Declarations

### Animal Studies

No animal studies were carried out by the authors for this article.
