# Supplementary material for: Inflow from a Cardiopulmonary Assist System to the Pulmonary Artery and Its Implications for Local Hemodynamics—a Computational Fluid Dynamics Study
Source: J Cardiovasc Transl Res. 2023 Jan 20;16(4):842–51. doi: 10.1007/s12265-022-10349-3 (PMC10480287; doi:10.1007/s12265-022-10349-3)
Supplement: Supplementary file 1 — Supplementary file1 Online Resource 1: OR_1_SupplementaryMaterial.pdf. The Supplementary Material contains information regarding meshing parameters, mesh convergence analysis and simulation setup as well as additional figures of the wall shear stress on the vessel wall. (PDF 465 KB) [file 12265_2022_10349_MOESM1_ESM.pdf]

Journal of Cardiovascular Translational Research

Supplementary Material

Inflow from a Cardiopulmonary Assist System to the Pulmonary Artery and its Implications for Local Hemodynamics – a Computational Fluid Dynamics Study

Kristin Hugenroth[1*], Felix Krooß[1], Flutura Hima[2], Lasse Strudthoff[1], Rüdger Kopp[3], Jutta Arens[1], Sebastian Kalverkamp[2], Ulrich Steinseifer[1], Michael Neidlin[1], Jan Spillner[2]

[1] Department of Cardiovascular Engineering, Institute of Applied Medical Engineering. Medical Faculty, RWTH Aachen University, Aachen, Germany

[2] Department of Thoracic and Cardiovascular Surgery, University Hospital, Medical Faculty, RWTH Aachen University, Aachen, Germany

[3] Department of Intensive Care Medicine and Intermediate Care, University Hospital, Medical Faculty, RWTH Aachen University, Aachen, Germany

* Corresponding author, e-mail address: kristin.hugenroth@rwth-aachen.de

S1: Meshing parameters

Maximum size: 1 mm
Curvature refinement: 15
Prism layers: 8
Initial height: 0.04 mm
Height ratio: 1.2

S2: Mesh convergence analysis

A mesh convergence analysis was conducted with the setup "S" (short jet path, central graft anastomosis) at 2.5 L/min graft flow to investigate the influence of the mesh size on the relevant results. Three meshes were generated: Mesh 1 (719,248 elements), Mesh 2 (944,969 elements) and Mesh 3 (1,146,952 elements).

The selected parameters for this analysis were area-averaged wall shear stress (ave. WSS), the flow rates at each outlet as well as the overall flow rate to side A and side B.

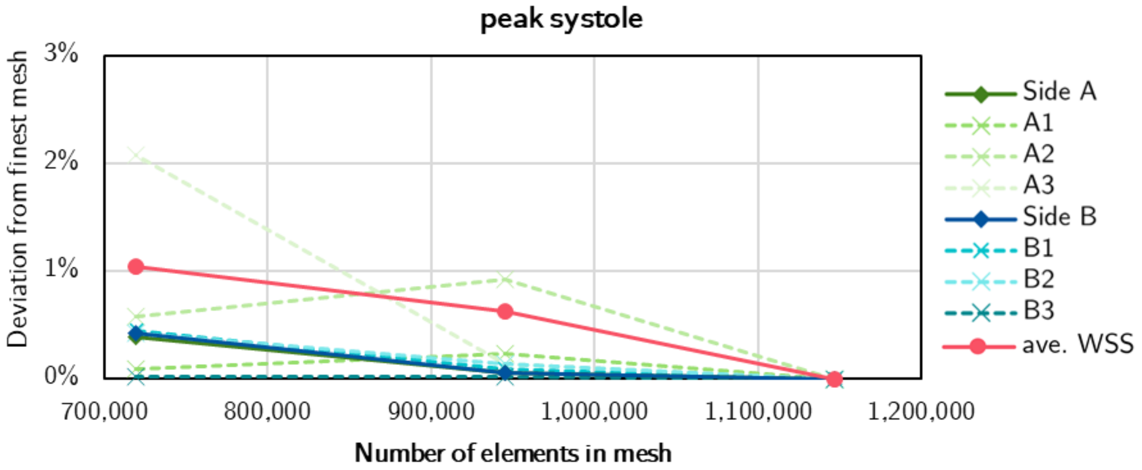

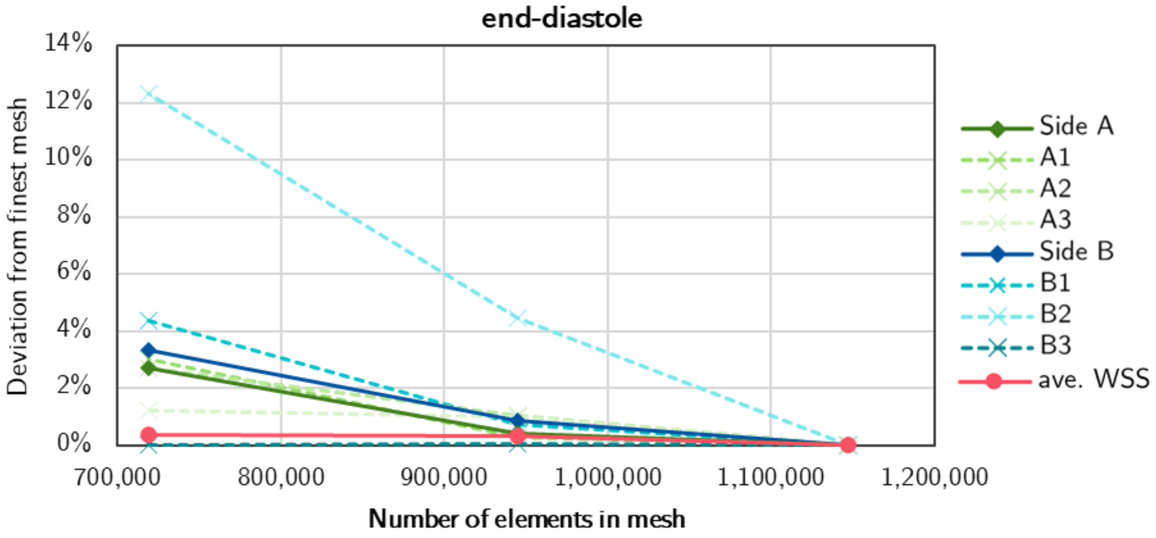

Figure S 1: Results of the mesh convergence analysis. Displayed are the deviation of the Mesh 1 (719,248 elements) and Mesh 2 (944,969 elements) from the finest Mesh 3 (1,146,952 elements). Upper graph: deviation at peak systole. Lower graph: deviation at end-diastole. "Side A" and "Side B" refer to the overall flows through the branches of the respective side; "A1", "A2", "A3", "B1", "B2", "B3" refer to the flow rates through the respective branches; "ave. WSS" refers to the area-averaged wall shear stress on the vessel wall.

The results are shown in Figure S 1. Based on these results, Mesh 2 was deemed suitable for the further study and used as a reference for setups "physiological", "M" and "L".

S3: Simulation setup

Table S 1 lists the boundary conditions for the simulation. Fig. S 2 illustrates the inflow boundary conditions from the right ventricle (RV). These were modeled using a lumped parameter model, which was adjusted to the data of one VV-ECMO patient on two different days (partial support and full support with increased heart rate).
The Reynolds numbers (Re) at in- and outlets were calculated by following equation (Reynolds number in pipe flow):

$$Re = \frac{v * \rho * D}{\eta}$$

with v being the velocity, ρ the blood density, D the vessel diameter and η the dynamic viscosity of the blood. As known from literature, 2300 can be assumed as the critical Reynolds number, above which a transition from laminar to turbulent flows can be expected in an unsteady setting like the presented one [1]. In cardiovascular flows, turbulent effects can be present below this threshold due to pulsation and deviation from the circular cross section shape [2]. A Reynolds number of 2300 is, for example, exceeded at the RV inlet at partial support (peak Re: 2676.6) and at the graft inlet at full support (Re: 3113.5). This leads to the assumption that a moderate level of turbulence can be expected at these boundaries and that the simulated PA flow is in the transitional range.

Table S 1 Boundary conditions for the simulation setups

Setup	Graft flow (L/min)	Average RV flow (L/min)	Peak RV flow velocity (m/s)	Heart rate (bpm)	Womersley number at RV inlet	Reynolds number at graft inlet	Turbulence intensity at graft inlet
Physiological PA	n/a	5.0	0.80	100	17.37	n/a	n/a
Partial support	2.5	2.9	0.46	101	17.46	1556.8	0%
Full support	5.0	0.8	0.11	114	18.55	3113.5	5%

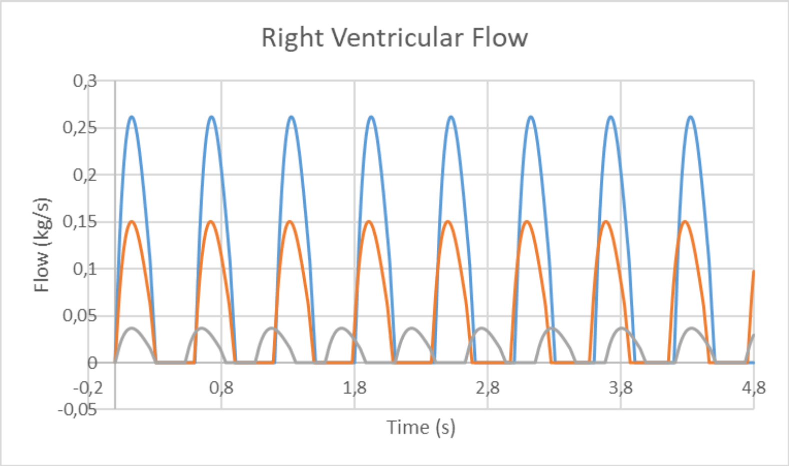

Fig. S 2 Right ventricular mass flow input for the simulations. Note the different heart rates, which reflect the different patient states.

S4: Average and maximum wall shear stress at distinct points in the cardiac cycle

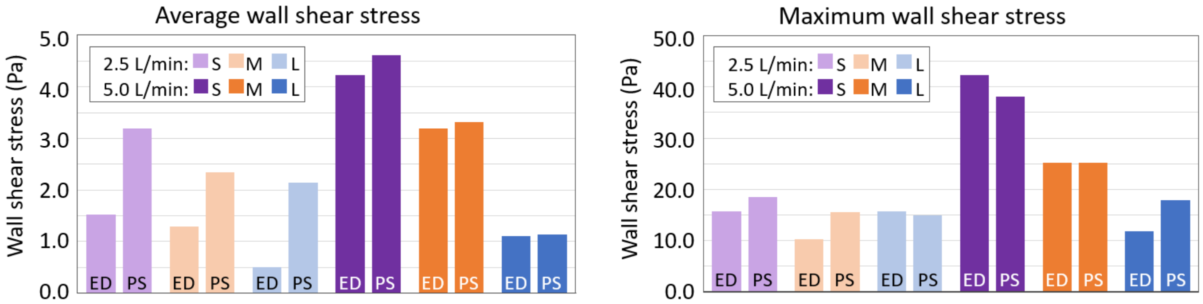

Fig. S 3 Wall shear stress on the pulmonary artery wall: average and maximum values at end-diastole (ED) and peak systole (PS) for 2.5 L/min and 5.0 L/min graft flow. Note the different y-axis scales. S: short jet path, M: medium jet path, L: long jet path. Note the differences in average WSS between ED and PS at 2.5 L/min graft flow.

S5: Wall shear stress at 2.5 L/min graft flow

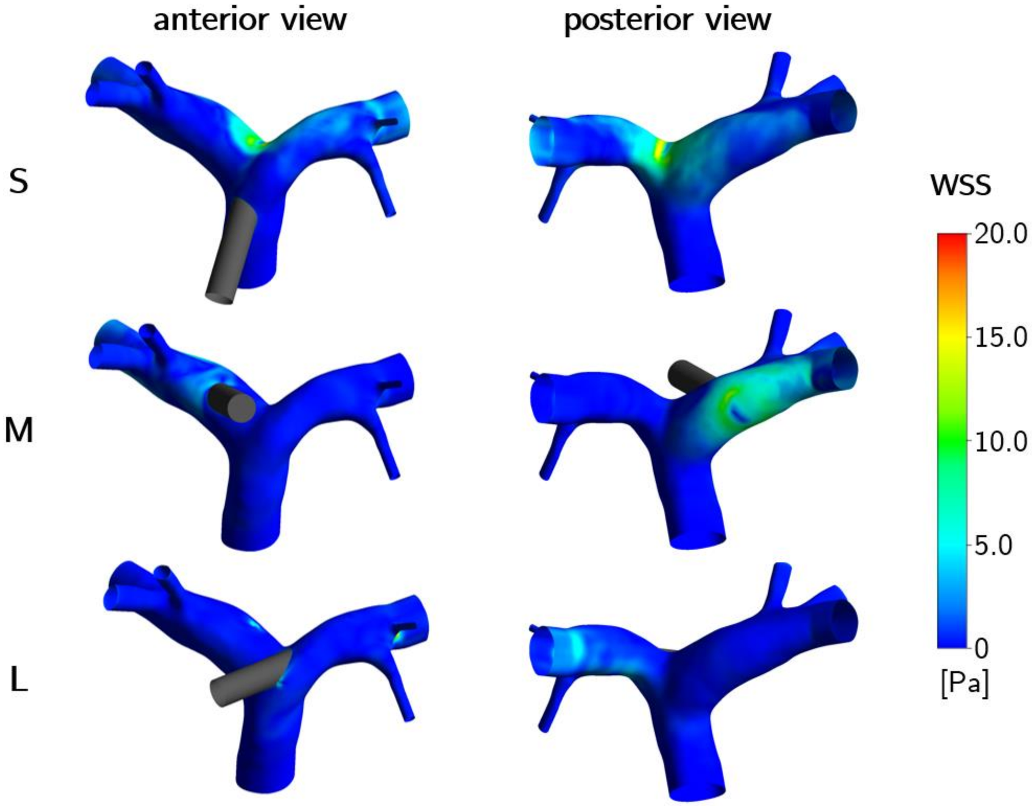

Fig. S 4 Wall shear stress (WSS) on the pulmonary artery wall at end-diastole and 2.5 L/min graft flow

S6: Wall shear stress at 5.0 L/min graft flow

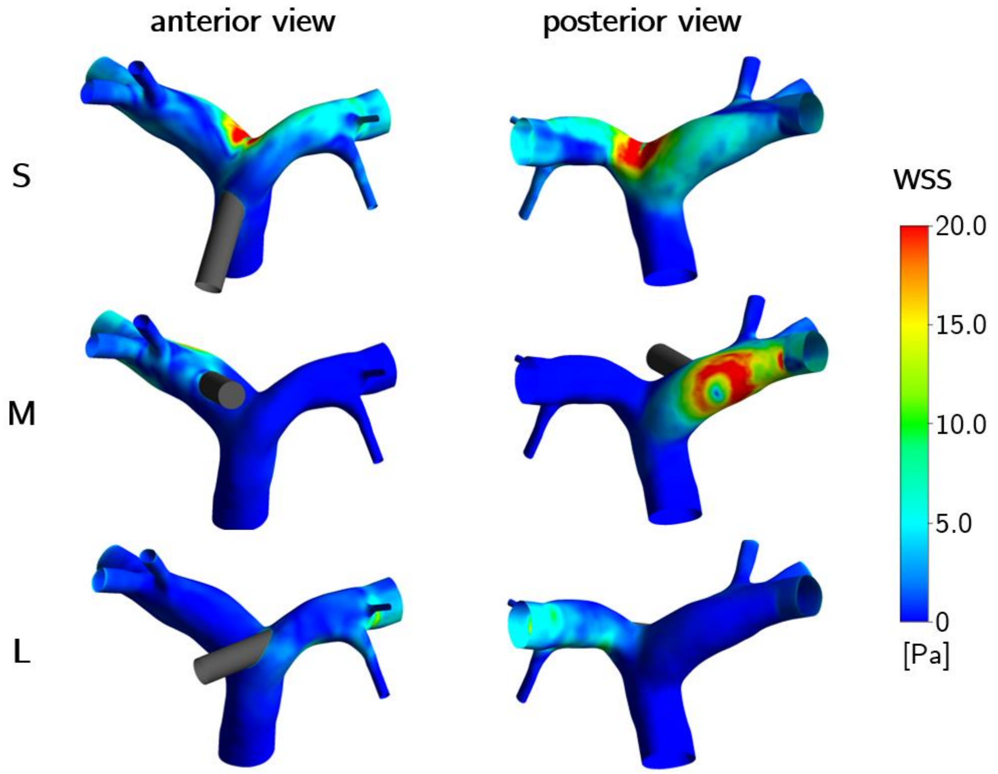

Fig. S 5 Wall shear stress (WSS) on the pulmonary artery wall at end-diastole and 5.0 L/min graft flow

References

1. Schlichting, H., & Gersten, K. (1997). *Grenzschicht-Theorie* (9th edn, Springer eBook Collection Computer Science and Engineering). Berlin, Heidelberg: Springer Berlin Heidelberg
2. Xu, D., Varshney, A., Ma, X., Song, B., Riedl, M., Avila, M., et al. (2020). Nonlinear hydrodynamic instability and turbulence in pulsatile flow. *Proceedings of the National Academy of Sciences of the United States of America,* 117(21), 11233–11239. 10.1073/pnas.1913716117
